# The COX10-AS1/miR-641/E2F6 Feedback Loop Is Involved in the Progression of Glioma

**DOI:** 10.3389/fonc.2021.648152

**Published:** 2021-07-26

**Authors:** Liang Liu, Xiaojian Li, Heming Wu, Yong Tang, Xiang Li, Yan Shi

**Affiliations:** Department of Neurosurgery, Nanjing First Hospital, Nanjing Medical University, Nanjing, China

**Keywords:** long non-coding RNA, COX10-AS1, E2F6, glioma, feedback loop

## Abstract

Glioma is the most common primary tumour of the central nervous system and is considered one of the greatest challenges for neurosurgery. Mounting evidence has shown that lncRNAs participate in various biological processes of tumours, including glioma. This study aimed to reveal the role and relevant mechanism of COX10-AS1 in glioma. The expression of COX10-AS1, miR-641 and E2F6 was measured by qRT-PCR and/or western blot. Clone formation assays, EdU assays, Transwell assays and tumour xenograft experiments were performed to evaluate the effects of COX10-AS1, miR-641 and E2F6 on glioma proliferation, migration and invasion. Luciferase reporter assays, RNA pull-down assays and ChIP assays were conducted to analyse the relationship among COX10-AS1, miR-641 and E2F6. We demonstrated that COX10-AS1 was upregulated in glioma tissues and cell lines, which was related to the grade of glioma and patient survival. Next, through functional assays, we found that COX10-AS1 influenced the proliferation, migration and invasion of glioma cell lines. Then, with the help of bioinformatics analysis, we confirmed that COX10-AS1 regulated glioma progress by acting as a sponge of miR-641 to regulate E2F6. Moreover, further study indicated that E2F6 could promote COX10-AS1 expression by binding to its promoter region. Taken together, the data indicated that COX10-AS1 acts as an oncogene in combination with COX10-AS1/miR-641/E2F6 in glioma, which may be beneficial to the diagnosis and treatment of glioma.

## Introduction

Glioma is the most common primary tumour in the central nervous system, accounting for approximately 60% of all intracranial primary tumours (1, 2). Glioblastoma multiforme (GBM) is the most lethal type of glioma according to the grade of malignancy, with an average survival time of 12 to 14 months and a five-year survival rate of only 4% to 5% (3, 4). The mortality rate of glioma has been stable at 4-5/100,000, ranking among the top 10 in tumour mortality (5). At present, the treatment of glioma is mainly by surgery, supplemented by radiotherapy and chemotherapy. However, because the tumour tissue is invasive and the boundary between the normal brain tissues (NBTs) is not clear, surgical resection is difficult (6). Glioma with a higher degree of malignancy is not sensitive to radiotherapy, and a high dose of radiation will cause normal brain tissue damage, so the clinical effective rate is only 50% (7). Most chemotherapy drugs cannot be used for treatment because they have difficulty crossing the blood-brain barrier. Overall, active research on the molecular mechanism of glioma is very important for the formulation of treatment strategies for glioma.

Current research has found that most sequences in the genome do not encode proteins and commonly known as non-coding RNAs (ncRNAs), which include circular RNAs (circRNAs), long non-coding RNAs (lncRNAs), and microRNAs (miRNAs) (8). Among them, lncRNAs are non-coding RNAs that do not have an open reading frame (ORF) and have a transcript length greater than 200 nucleotides (9). Initially, researchers found that the expression of many lncRNAs was dysregulated in a variety of tumours, including gliomas and that the expression levels of some lncRNAs could be used as prognostic indicators (10, 11). With further studies, researchers have found that lncRNAs can affect tumour progression by their involvement in a variety of cellular processes, such as proliferation, migration, invasion, autophagy, and epithelial-mesenchymal transition (EMT) (12–14). COX10 antisense RNA 1 (COX10-AS1) is a non-coding transcript located on chromosome 17 (14029292-14069458, complement). Till now, little is known about the role of COX10-AS1 in human diseases. Feng et al. reported that compared with that in healthy oral mucosa, COX10-AS1 was upregulated in oral squamous cell carcinoma tissues (15). Luan et al. found that COX10-AS1 was associated to autophagy and the prognosis of glioma patients (16). However, the regulatory network of COX10-AS1 in the progression of glioma has not been elucidated.

In our present study, we analysed the expression of COX10-AS1 in a public database and in clinical specimens. Consistent with the previous studies, COX10-AS1 was upregulated in glioma tissues and cell lines. Next, gain/loss-of-function assays indicated that COX10-AS1 participated in the proliferation, migration and invasion of glioma cells. Then, with the help of bioinformatics tools and related assays, we demonstrated that COX10-AS1 promoted glioma progression by sponging miR-641 to regulate E2F6. More interestingly, we found that E2F6, a well-known transcription factor (TF), could regulate COX10-AS1 expression by directly binding to its promoter region. By using rescue assays, we concluded that COX10-AS1/miR-641/E2F6 formed a positive feedback loop in glioma progression, which may provide a theoretical basis for the development of new treatment strategies for glioma.

## Materials and Methods

### Clinical Specimens

Thirty glioma tissues and paired adjacent normal brain tissues were collected from patients who were diagnosed with glioma at Nanjing First Hospital. The tissues were frozen in liquid nitrogen and stored at -80°C immediately after surgical resection. All the participants signed written informed consent forms. This research was approved by the Ethics Committee of the Nanjing First Hospital.

### Cell Lines

Glioma cell lines (U87, U118, T98G, A172 and LN229) were obtained from Procell (Wuhan, China). The normal human astrocytes (NHAs) obtained from JENNIO Biological Technology (Guangzhou, China). All six cell lines were cultured in Dulbecco’s modified Eagle’s medium (DMEM, Gibco, NY, USA, Cat. No. 11965092) containing 10% foetal bovine serum (FBS, ScienCell, LA, USA, Cat. No. 0500) and were incubated in an atmosphere containing 5% CO_2_ at 37°C.

### Interfering Nucleotide Transfection

The chemically synthesized oligonucleotides used in this study were designed and constructed by GenePharma (Shanghai, China). The transfections were performed using Lipofectamine 3000 (Invitrogen, Carlsbad, CA, USA, Cat. No. 2185325) according to the manufacturer’s instructions. The interfering nucleotide used in this study were shown in **Table S1**.

### RNA Isolation and Quantitative Real-Time Polymerase Chain Reaction (qRT-PCR)

RNA extraction and qRT-PCR were performed as described previously (17). Total RNA was extracted from clinical tissue and cells by TRIzol reagent (Invitrogen, Carlsbad, CA, USA, Cat. No. 15596018). qRT-PCR was conducted using an ABI Prism 7700 Sequence Detection System (Applied Biosystems, Thermo Fisher Scientific, MA, USA) according to the manufacturer’s instructions. β-actin was used as an internal control. Relative expression levels of COX10-AS1, miR-641 and E2F6 were measured using the 2^–ΔΔCt^ method. The primers used in this study were shown in **Table S2**.

### Nuclear and Cytoplasmic RNA Fraction Isolation

Nuclear and cytoplasmic RNA were isolated from each fraction using a Nuclear/Cytosol Fractionation Kit (BioVision, San Francisc, CA, USA, Cat. No. XY-K266-25) following the manufacturers’ instructions. U6 and 18S were used as a nuclear control and cytoplasmic control, respectively.

### Western Blot

Total protein was extracted from the cells by using RIPA buffer (KenGEN, Shanghai, China, Cat. No. KGB704), and the protein concentrations were quantified by using a BCA Protein Assay Kit (Beyotime, Shanghai, China, Cat. No. P0012S). The steps were the same as those described in our previous study (18). The primary antibodies used in this assay were purchased from Abcam (Cambridge, UK, Cat. No. ab53061 & ab8227).

### Fluorescence *In Situ* Hybridization (FISH)

FISH assays were performed with a FISH Kit (GenePharma, Shanghai, China, Cat. No. F11202) according to the manufacturer’s instructions. The probe used in this study was synthesized and purchased from GenePharma (Shanghai, China). Cells were fixed in 4% paraformaldehyde for 20 min. Next, the cells were preincubated and incubated at 37°C for 30 min with PBS and hybridization solution, respectively. After that, cell nuclei were stained by using DAPI (4′,6-diamidino-2-phenylindole, Beyotime, Jiangsu, China, Cat. No. C1002). The images were captured using a fluorescence microscope (Zeiss, Germany).

### RNA Pull-Down Assay

Biotinylated miR-641 and the corresponding mutant/negative control were synthesized and purchased from GenePharma (Shanghai, China). The oligonucleotides were transfected into U87 and LN229 cells using Lipofectamine 3000 (Invitrogen). Forty-eight hours later, the cell lysates were incubated with M-280 streptavidin magnetic beads (Invitrogen, Cat. No. 20164). qRT-PCR was used to detect the expression of COX10-AS1.

### Chromatin Immunoprecipitation (ChIP)

ChIP assays were performed by using a ChIP Kit (Magna, Millipore, Bedford, MA, USA, Cat. No. 17-371). The collected cells were cross-linked by formaldehyde, and the reaction was terminated by glycine. After incubation with lysis buffer for 30 min, the cells were sheared by sonication and centrifuged. Then, the DNA–protein complexes were immunoprecipitated using antibodies (anti-E2F6, Abnova, China, Cat No. H00001876-PW1 and lgG, Proteintech, USA, Cat. No. 66360-3-Ig). qRT-PCR was used to detect the purified DNA.

### Luciferase Reporter Assay

The fragments of COX10-AS1 or E2F6 containing the miR-641 binding sites (wild type, WT) and negative controls (mutant, MUT) were amplified and cloned into the pGL3 vector (Promega, Madison, WI, USA, Cat No. E1751). Then, U87 and LN229 cells were transfected with miR-641 mimics or controls by using Lipofectamine 3000 (Invitrogen) in accordance with the manufacturers’ instructions. Forty-eight hours later, the luciferase activity of the cells was measured by the Dual-Luciferase Reporter Assay System (Promega, WI, USA).

### Clone Formation Assay

300 cells were grown in culture plates (60 mm, Corning, NY, USA) and maintained in DMEM containing 10% FBS. 14 d later, cells were fixed with paraformaldehyde (4%) for 20 min. Then stained with 0.1% crystal violet for 15 min. Finally, visible colonies were counted.

### 5-Ethynyl-20-deoxyuridine (EdU) Assay

EdU assays were performed with an EdU Cell Proliferation Kit (RiboBio, Guangzhou, China, Cat No. 10310-3). The protocol was the same as that described in our previous study (19). A fluorescence microscope (Olympus, Japan) was used to acquire the images.

### Transwell Assays

Transwell assays were performed as described previously (18). Matrigel (1:9 dilution, BD, NJ, USA, Cat No. 356234) was used to precoat the upper chamber for Transwell invasion assays. Forty-eight hours later, the cells were fixed, strained and counted in turn.

### Immunohistochemistry (IHC)

Immunohistochemistry was performed as described previously (17). Paraffin-embedded tissues were incubated with a primary antibody against E2F6 (1:200, Abcam, Cambridge, UK, Cat. No. ab53061) or Ki-67 (1:200, CST, MA, USA, Cat. No. 9449) at 4°C for 12 h. Then, the tissues were incubated with a secondary antibody (1:1000, Boster, Wuhan, Hubei, China, Cat. No. BM3895 & BA1082) at room temperature for 1 h. After incubation with ABC-peroxidase at room temperature for 1 h, the tissues were stained with diaminobenzidine for 5 min. The images were captured using a fluorescence microscope (Olympus, Japan).

### Terminal Deoxynucleotidyl Transfer-Mediated dUTP Nick end Labelling Staining (TUNEL)

The tissues were deparaffinized in xylene, followed by washing in alcohol. Apoptotic cells were detected by a TUNEL Kit (Roche, Mannheim, Germany, Cat. No.11767291910) according to the manufacturer’s instructions.

### Intracranial Tumour Mouse Model

Forty male nude mice purchased from the Chinese Academy of Sciences were randomly divided into four groups (10 mice per group). LN229 cells (2×10^6^) stably expressing luciferase were transfected with sh-COX10-AS1, sh-E2F6 and the corresponding negative controls. Next, the cells were intracranially injected into the frontal lobe of nude mice. A bioluminescence imaging system was used to quantify the volumes of the tumours formed intracranially every ten days after implantation. The Living Images software package (Caliper Life Science, Waltham, MA, USA) was used to determine the integrated flux of photons (photons/s). Mouse survival data were recorded in detail until all the mice died. Brain tissue and the tumour tissue that formed in the brain were removed intact for immunohistochemical analysis and other experiments. The animal experiments were approved by the Institutional Animal Care and Use Committee of Nanjing First Hospital.

### Statistical Analysis

SPSS 20.0 (IBM, NY, USA) was used to analyse the data. The data are expressed as the means ± standard deviation (SD). Student’s t-test or one-way ANOVA was used to evaluate the differences between groups. Overall survival was evaluated by the Kaplan–Meier method. P<0.05 indicates statistical significance. All experiments were carried out three times independently.

## Results

### COX10-AS1 Was Upregulated in Glioma Tissues and Cell Lines

**Figure 1:**
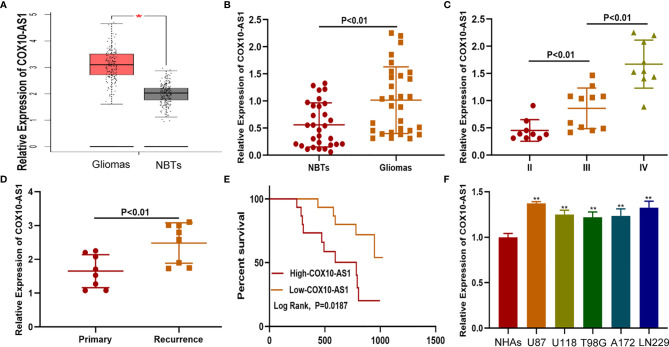
COX10-AS1 is upregulated in glioma tissues and cell lines. **(A)** The relative expression of COX10-AS1 in GEPIA. **(B)** The relative expression of COX10-AS1 in clinical specimens. **(C)** The relative expression of COX10-AS1 in different grade of glioma. **(D)** The relative expression of COX10-AS1 in clinical specimens from patients with primary and recurrent glioma. **(E)** Kaplan–Meier curve analysis of the correlation between COX10-AS1 expression and overall survival from our clinical data. **(F)** The relative expression of COX10-AS1 in NHAs and glioma cell lines. *P < 0.05, **P < 0.01.

To detect the expression of COX10-AS1 in glioma, online databases (GEPIA, http://gepia.cancer-pku.cn/index.html) were utilized. The data from GEPIA showed that the expression of COX10-AS1 in glioma tissues was higher than that in normal brain tissues (**Figure 1A**). We also explored COX10-AS1 expression in clinical species collected during surgery, and the aresult was similarly to that of the online databases (**Figures 1B, C**). To evaluate whether COX10-AS1 can be used as an indicator to predict the recurrence of glioma, the expression level of COX10-AS1 in the tissues of primary and recurrent glioma was detected. Interestingly, the results showed that COX10-AS1 was upregulated in recurrent glioma, suggesting that COX10-AS1 is closely related to glioma recurrence (**Figure 1D**). Moreover, clinical data of 30 glioma patients was collected and Kaplan-Meier curves indicated that glioma patients with high COX10-AS1 expression had poorer survival than those with low COX10-AS1 expression (**Figure 1E**). The correlation of clinicopathological characteristics between COX10-AS1 and glioma patients was shown in **Table 1**. In addition, we measured COX10-AS1 expression in normal human astrocytes and glioma cell lines. The results showed that the COX10-AS1 levels in glioma cell lines were much higher than those in NHAs, especially in U87 and LN229 cells (**Figure 1F**). These findings suggest that COX10-AS1 is closely related to the malignant progression of glioma.

**Table 1:** Correlation of clinicopathological characteristics between COX10-AS11 and glioma patients.

Clinicopathologic data	Case (n)	COX10-AS1 expression	P value
Gender			P = 0.8619
Male	17	1.0240 ± 0.5938	
Female	13	0.9859 ± 0.5361	
Age (years)			P = 0.6785
<60	11	0.9520 ± 0.6392	
≥60	19	1.0511 ± 0.5804	
Tumor diameter (cm)			P < 0.05
<3	13	0.7588 ± 0.4898	
≥3	17	1.2105 ± 0.6107	
WHO classification			P < 0.01
I+II	10	0.5304 ± 0.2749	
III+IV	20	1.2460 ± 0.5278	

### COX10-AS1 Promotes Glioma Proliferation, Migration and Invasion *In Vitro* and Glioma Growth *In Vivo*

**Figure 2:**
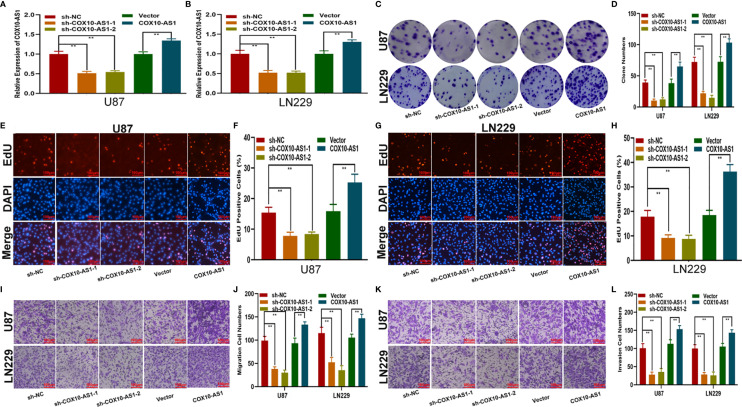
COX10-AS1 promotes glioma proliferation, migration and invasion *in vitro.* **(A, B)** Relative expression of COX10-AS1 in U87 and LN229 cells transfected with sh-COX10-AS1 (including sh-COX10-AS1-1, sh-COX10-AS1-2), COX10-AS1 plasmid or the corresponding negative control, as measured by qRT-PCR. **(C, D)** The proliferation of U87 and LN229 cells transfected with sh-COX10-AS1, COX10-AS1 plasmid or the corresponding negative control, as measured by clone formation assays. **(E–H)** The proliferation of U87 and LN229 cells transfected with sh-COX10-AS1, COX10-AS1 plasmid or the corresponding negative control, as measured by EdU assays. **(I, J)** The migration of U87 and LN229 cells transfected with sh-COX10-AS1, COX10-AS1 plasmid or the corresponding negative control, as measured by Transwell assays. **(K, L)** The invasion of U87 and LN229 cells transfected with sh-COX10-AS1, COX10-AS1 plasmid or the corresponding negative control, as measured by Transwell assays. **P < 0.01.

**Figure 3:**
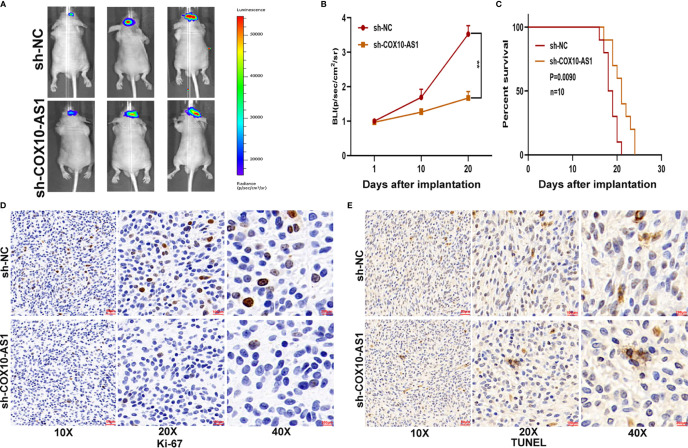
COX10-AS1 promotes glioma growth *in vivo*. **(A, B)** The bioluminescent images of the tumours formed in the brains of nude mice were acquired at days 1, 10 and 20 after implantation. **(C)** Overall survival was compared between the sh-COX10-AS1 and sh-NC groups by Kaplan-Meier survival curves. **(D)** Immunohistochemistry for Ki-67 in the sh-COX10-AS1 and sh-NC groups. **(E)** TUNEL staining in the sh-COX10-AS1 and sh-NC groups. **P < 0.01.

To study the relationship between COX10-AS1 and the malignant progression of glioma, we transfected short hairpin RNAs targeting COX10-AS1 (sh-COX10-AS1-1 and sh-COX10-AS1-2), a COX10-AS1 expression plasmid (COX10-AS1) and corresponding controls into U87 and LN229 cells. The efficiency of these chemosynthetic sequences was verified by qRT-PCR (**Figures 2A, B**). Next, clone formation assays and EdU assays revealed that silencing COX10-AS1 decreased the proliferation ability of U87 and LN229 cells, while upregulation of COX10-AS1 had the opposite effect (**Figures 2C–H**). Transwell assays illustrated that downregulation of COX10-AS1 reduced cell migration and invasion, whereas upregulation of COX10-AS1 increased the migration and invasion of U87 and LN229 cells (**Figures 2I–L**). Furthermore, we injected LN229 cells transfected with a fluorescent lentivirus expressing sh-COX10-AS1 (including sh-COX10-AS1-1 and sh-COX10-AS1-2) or the corresponding controls into the brains of nude mice. In vivo imaging of the mice was performed on the indicated days (1 d, 10 d and 20 d) after implantation. The results showed that tumour growth was obviously inhibited after COX10-AS1 silencing (**Figures 3A, B**). Furthermore, we found that the mice in the sh-COX10-AS1 group had better survival than those in the negative group (**Figure 3C**). In addition, immunohistochemistry assays showed that Ki-67 was downregulated and TUNEL staining was upregulated in tumour tissues from mice in the sh-COX10-AS1 group (**Figures 3D, E**). These results suggest that COX10-AS1 exhibits important functions in glioma.

### COX10-AS1 Acts as a Sponge for miR-641

**Figure 4:**
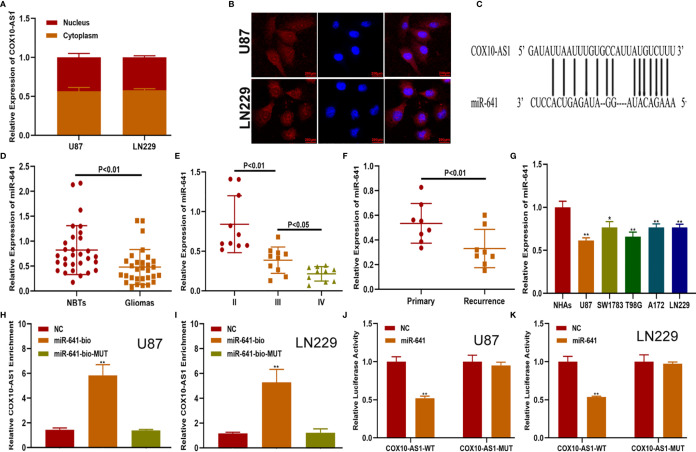
COX10-AS1 acts as a sponge for miR-641. **(A)** RT-qPCR assays in nuclear and cytoplasmic RNA fractions detected the COX10-AS1 level in the cytoplasm and nucleus. **(B)** The distribution of COX10-AS1 in U87 and LN229 cells was evaluated by FISH assays. **(C)** The putative binding sites of miR-641 on COX10-AS1 were predicted by an online database. **(D)** The relative expression of miR-641 in clinical specimens. **(E)** The relative expression of miR-641 in different grade of glioma. **(F)** The relative expression of miR-641 in primary and recurrent glioma clinical specimens. **(G)** The relative expression of miR-641 in NHAs and glioma cell lines. **(H, I)** U87 and LN229 cells were assayed by biotin-based pull-down after transfection with biotin-labelled miR-641. **(J, K)** Luciferase reporter assay indicated that miR-641 reduced the luciferase activity of COX10-AS1-WT but not COX10-AS1-MUT. *P < 0.05, **P < 0.01.

There are several mechanisms by which lncRNAs affect tumour progression, of which their role as competing endogenous RNAs (ceRNAs) is an important one (20). By this mechanism lncRNAs can act as sponges for miRNAs to regulate targeted message RNAs (mRNAs), which can promote or suppress tumour progression. To reveal the underlying mechanism of COX10-AS1 in glioma, we measured the expression of COX10-AS1 at the subcellular level. The results of qRT-PCR and FISH showed that COX10-AS1 was localized in both the cytoplasm and the nucleus (**Figures 4A, B**), indicating that COX10-AS1 is likely to exert its function of regulation of tumour progression by acting as a ceRNA. To determine the targeted miRNA of COX10-AS1, bioinformatics prediction was performed with StarBase (http://starbase.sysu.edu.cn/), and miR-641 greatly aroused our interest (**Figure 4C**). Next, we detected the expression of miR-641 in clinical specimens and found that miR-641 was higher in normal brain tissues than in glioma tissues (**Figures 4D, E**). Compared with primary glioma tissues, miR-641 expression in recurrent glioma tissues was lower (**Figure 4F**). Similarly, miR-641 was higher in NHAs than that in glioma cell lines (**Figure 4G**). Moreover, RNA pull-down assays showed that COX10-AS1 was pulled down by miR-641 in both U87 and LN229 cells (**Figures 4H, I**). Moreover, luciferase reporter assays indicated that miR-641 could decrease the luciferase activity of COX10-AS1-WT but not COX10-AS1-MUT (**Figures 4J, K**). Overall, we concluded that COX10-AS1 acts as a sponge for miR-641 in glioma cells.

### The Effect of COX10-AS1 on Glioma Is Partially Mediated by miR-641

**Figure 5:**
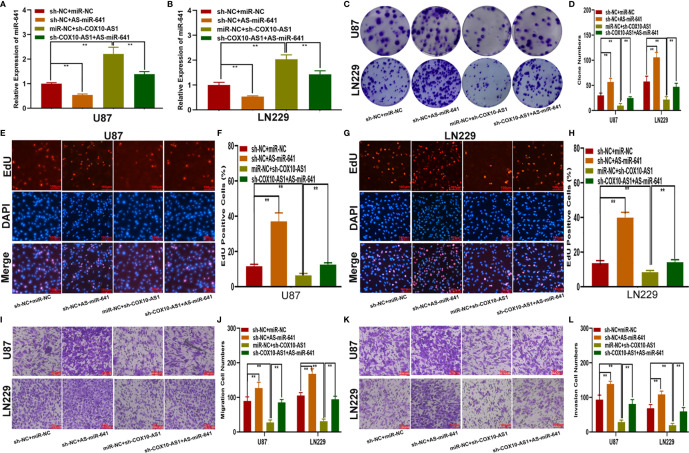
The effect of COX10-AS1 on glioma is partially mediated by miR-641. **(A, B)** The relative expression of miR-641 in U87 and LN229 transfected with miR-641 inhibitor, sh-COX10-AS1 or the corresponding controls, as measured by qRT-PCR. **(C, D)** The proliferation of U87 and LN229 cells transfected with miR-641 inhibitor, sh-COX10-AS1 or the corresponding controls, as measured by clone formation assays. **(E–H)** The proliferation of U87 and LN229 cells transfected with miR-641 inhibitor, sh-COX10-AS1 or the corresponding controls, as measured by EdU assays. **(I, J)** The migration of U87 and LN229 cells transfected with miR-641 inhibitor, sh-COX10-AS1 or the corresponding controls, as measured by Transwell assays. **(K, L)** The invasion of U87 and LN229 cells transfected with miR-641 inhibitor, sh-COX10-AS1 or the corresponding controls, as measured by Transwell assays. **P < 0.01.

To explore the function of miR-641 in COX10-AS1 promoting the malignant progression of glioma, four cell models were constructed with miR-641 inhibitor, sh-COX10-AS1 and the corresponding negative controls. qRT-PCR showed that sh-COX10-AS1 obviously upregulated miR-641 (**Figures 5A, B**). Clone formation assays and EdU assays indicated that the miR-641 inhibitor could promote the proliferation of glioma cells, and the promoting effect could be reversed by sh-COX10-AS1 (**Figures 5C–H**). Similarly, the Transwell assays showed that miR-641 inhibitor could facilitate the migration and invasion of glioma cells, and the facilitating effect was partially rescued by sh-COX10-AS1 (**Figures 5I–L**). These functional assays indicated that miR-641 plays an important role in the carcinogenic effect of COX10-AS1 on glioma progression.

### E2F6 is the Functional Target of the COX10-AS1/miR-641 Axis

**Figure 6:**
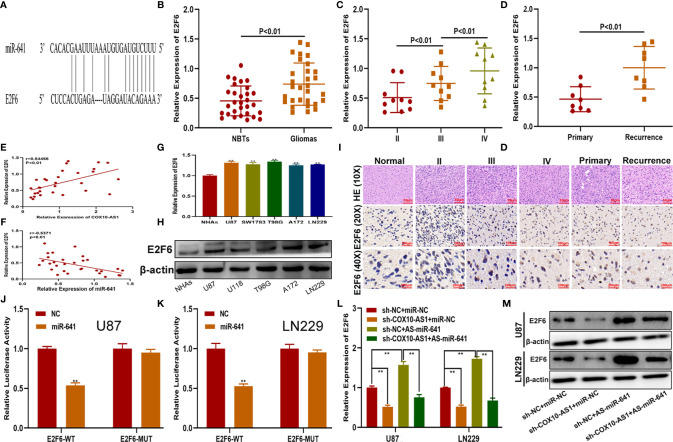
E2F6 is the functional target of the COX10-AS1/miR-641 axis. **(A)** The putative binding sites of miR-641 on E2F6 were predicted by an online database. **(B)** The relative expression of E2F6 in clinical specimen. **(C)** The relative expression of E2F6 in different grade of glioma. **(D)** The relative expression of E2F6 in primary and recurrent glioma. **(E)** Pearson’s correlation analysis of COX10-AS1 expression and E2F6 expression in clinical specimens. **(F)** Pearson’s correlation analysis of miR-641 expression and E2F6 expression in clinical specimens. **(G, H)** The relative expression of E2F6 in NHAs and glioma cell lines measured by qRT-PCR and western blot. **(I)** The expression level of E2F6 in different grades of glioma detected by immunohistochemistry. **(J, K)** Luciferase reporter assay indicated that miR-641 reduced the luciferase activity of E2F6-WT but not E2F6-MUT. **(L, M)** The relative expression of E2F6 in U87 and LN229 cells transfected with sh-COX10-AS1, miR-641 inhibitor or sh-COX10-AS1 together with miR-641 inhibitor, as measured by qRT-PCR and western blot. **P < 0.01.

To verify the functional target of the COX10-AS1/miR-641 axis, we searched the StarBase database. Considering the database results and the expression data from our clinical specimens, we chose E2F6 for further study (**Figure 6A**). qRT-PCR showed that the expression of E2F6 in glioma tissues was higher than that in normal brain tissues and was correlated with the recurrence of glioma (**Figures 6B–D**). Pearson’s correlation analysis indicated that there was a significant correlation between E2F6 and COX10-AS1/miR-641 (**Figures 6E, F**). We explored E2F6 expression in NHAs and glioma cell lines by qRT-PCR and western blot as well. The results demonstrated that E2F6 was higher in glioma cells than in NHAs (**Figures 6G, H**). Immunohistochemistry assays showed the similar results (**Figure 6I**). Luciferase reporter assays indicated that, compared with that of E2F6-MUT, miR-641 obviously decreased the luciferase activity of E2F6-WT (**Figures 6J, K**). Otherwise, qRT-PCR and western blot assays showed that miR-641 inhibitor could upregulate the expression of E2F6 and that the promoting effect could be inhibited by sh-COX10-AS1 (**Figures 6L, M**). Thus, we concluded that E2F6 is the functional target of the COX10-AS1/miR-641 axis.

### E2F6 Promotes Glioma Proliferation, Migration and Invasion *In Vitro* and Glioma Growth *In Vivo*

**Figure 7:**
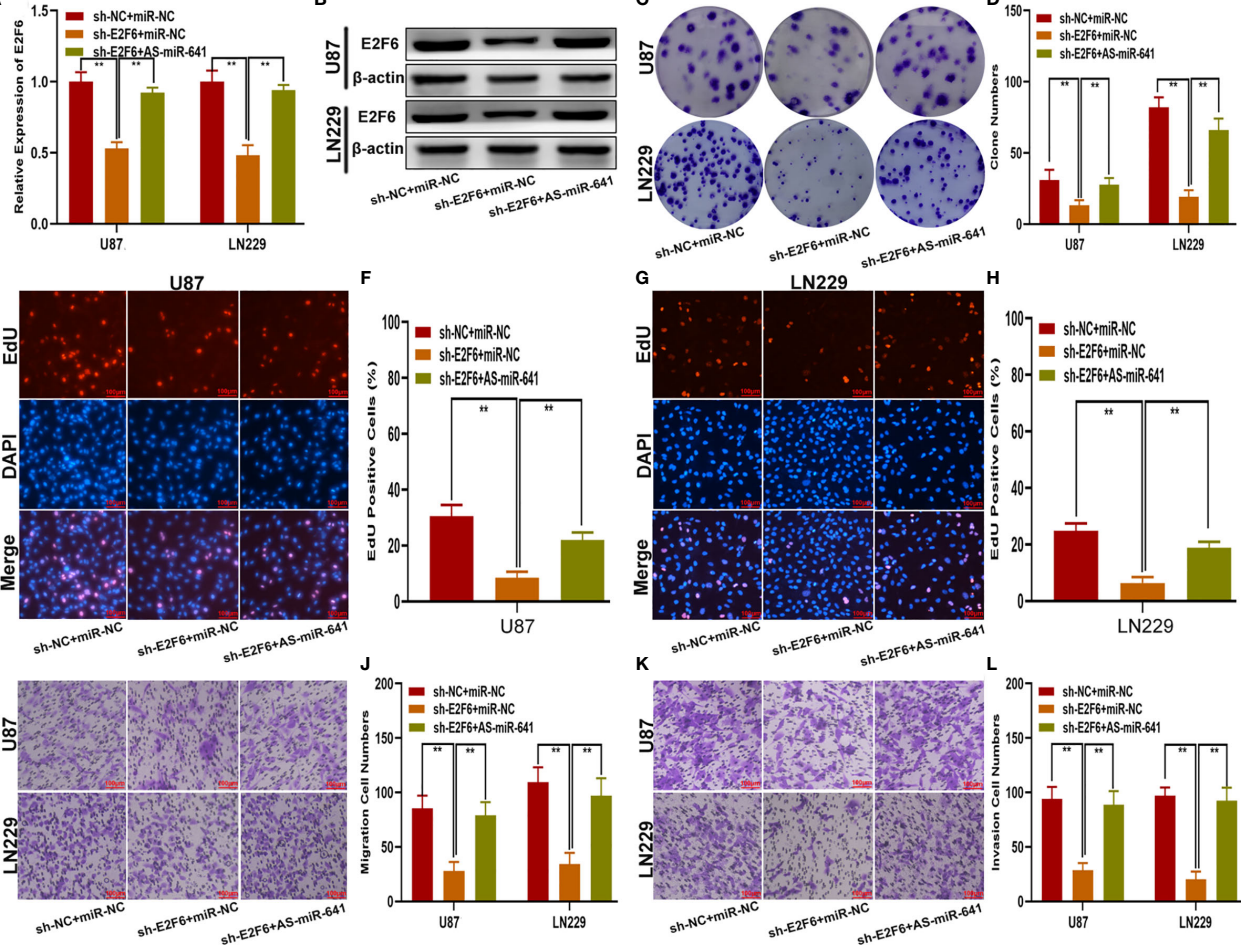
E2F6 promotes glioma proliferation, migration and invasion *in vitro.* **(A, B)** The relative expression of E2F6 in U87 and LN229 cells transfected with sh-E2F6 or sh-E2F6 together with miR-641 inhibitor, as measured by qRT-PCR and western blot. **(C, D)** The proliferation of U87 and LN229 cells transfected with sh-E2F6 or sh-E2F6 together with miR-641 inhibitor, as measured by clone formation assays. **(E–H)** The proliferation of U87 and LN229 cells transfected with sh-E2F6 or sh-E2F6 together with miR-641 inhibitor, as measured by EdU assays. **(I, J)** The migration of U87 and LN229 cells transfected with sh-E2F6 or sh-E2F6 together with miR-641 inhibitor, as measured by Transwell assays. **(K, L)** The invasion of U87 and LN229 cells transfected with sh-E2F6 or sh-E2F6 together with miR-641 inhibitor, as measured by Transwell assays. **P < 0.01.

**Figure 8:**
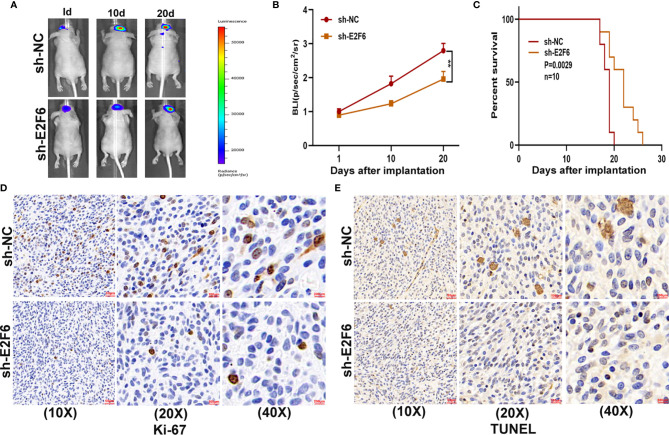
E2F6 promotes glioma growth *in vivo*. **(A, B)** The bioluminescent images of the tumours formed in the brains of nude mice were acquired at days 1, 10 and 20 after implantation. **(C)** Overall survival was compared between the sh-E2F6 and sh-NC groups by Kaplan-Meier survival curves. **(D)** Immunohistochemistry for Ki-67 in the sh-E2F6 and sh-NC groups. **(E)** Immunohistochemistry for TUNEL staining in the sh-E2F6 and sh-NC groups.

To explore whether E2F6 regulated the function of miR-641, three cell models were constructed with sh-E2F6, miR-641 inhibitor and the negative control. qRT-PCR and western blot assays confirmed that miR-641 inhibitor reversed the E2F6 downregulation caused by sh-E2F6 (**Figures 7A, B**). Clone formation assays and EdU assays demonstrated that sh-E2F6 could inhibit the proliferation of glioma cells and that the inhibitory effect could be rescued by miR-641 inhibitor (**Figures 7C–H**). Transwell assays showed a similar result whereby sh-E2F6 could inhibit the migration and invasion of glioma cells, and the inhibitory effect could be partly reversed by miR-641 inhibitor (**Figures 7I–L**). In addition, we explored the effect of E2F6 on glioma growth *in vivo*. The results demonstrated that downregulation of E2F6 inhibited tumour growth and extended the survival time (**Figures 8A–C**). Moreover, Ki-67 and TUNEL staining suggested that E2F6 was involved in the progression of glioma growth (**Figures 8D, E**).

### E2F6 Regulates COX10-AS1 Expression by Binding to Its Promoter Region

**Figure 9:**
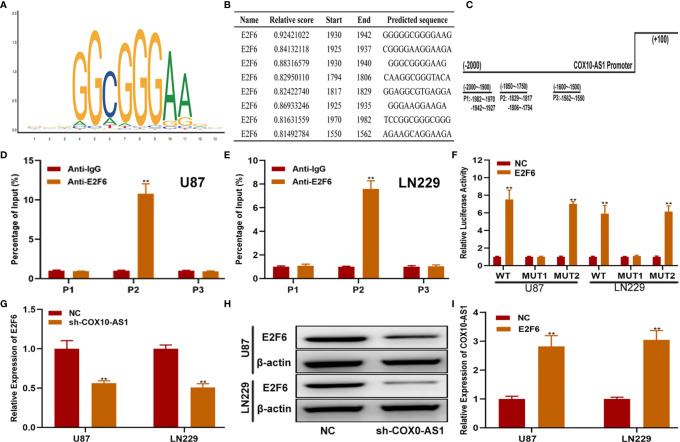
E2F6 regulates COX10-AS1 expression by binding to its promoter region. **(A)** The binding motif of E2F6 determined from JASPAR. **(B, C)** The top five binding sites in the COX10-AS1promoter and their corresponding three counterparts in the promoter region are shown. **(D, E)** ChIP assays were performed to reveal the affinity of E2F6 to the COX10-AS1 promoter in U87 and LN229 cells. **(F)** Luciferase reporter assays were used to locate the binding sequences of E2F6 to the COX10-AS1 promoter. **(G, H)** The expression level of E2F6 in glioma cell lines transfected with sh-COX10-AS1 or sh-NC, as measured by qRT-PCR and western blot. **(I)** The expression level of COX10-AS1 in glioma cell lines transfected with E2F6 plasmid or NC, as measured by qRT-PCR. **P < 0.01.

To further explain the regulatory relationship among COX10-AS1, miR-641 and E2F6, we tested whether E2F6 could regulate COX10-AS1. First, we searched the promoter sequence of COX10-AS1 *via* UCSC (https://genome.ucsc.edu/). With the help of the JASPAR website (http://jaspar.genereg.net/), we found that there are sites on E2F6 that could bind to the promoter sequence of COX10-AS1 (**Figures 9A–C**). By ChIP assays, we found that the affinity of the COX10-AS1 promoter (P2) to E2F6 was stronger than that to IgG (**Figures 9D, E**). To validate the effectiveness of the binding sites (including two sites), luciferase reporter assays was conducted, and the results indicated that the promoter activity of the predicted sites (-1804 ~ -1794) was enhanced significantly by E2F6 (**Figure 9F**). Finally, we found a positive correlation between E2F6 and COX10-AS1 by qRT-PCR and/or western blot (**Figures 9G–I**). Overall, we concluded that COX10-AS1/miR-641/E2F6 formed a positive feedback loop to regulate glioma progression.

## Discussion

In 1976, E Zuckerkandl reported that ncRNAs may exert their function by regulating the transcription process, which caused a paradigm shift because researchers generally believed that ncRNAs were useless and were therefore called “rubbish” (21). Over time, mounting advances in ncRNAs research have shown that dysregulation of ncRNAs is closely related to human diseases, including cancers. For instance, miR-452 regulates the progression of gastric cancer by targeting EPB41L3 (22); SNHG1 (small nucleolar RNA host gene 1) promotes the malignant development of glioma by acting as a sponge of miR-194 (19); and Circ_0079593 may function as a prognostic indicator in glioma (23). LncRNAs, an important class of ncRNAs longer than 200 nucleotides, have aroused great interest among researchers. Till now, multiple lines of research have suggested that lncRNAs have critical effects on tumour processes, such as proliferation, apoptosis, and invasion (24–26). COX10-AS1, a lncRNA transcript from chromosome 17, has been shown to be related to the development of human cancers. For example, Lu et al. demonstrated that COX10-AS1 is dysregulated in oral squamous cell carcinoma (15) and Luan et al. reported that COX10-AS1 is involved in the development of glioma via regulation of autophagy (16). Although existing research has indicated that COX10-AS1 is closely related to glioma, the underlying mechanism is elusive and has not been completely clarified. In the current study, through the public databases GEPIA, we determined that COX10-AS1 was upregulated in glioma. Consistent with the results from the public database, we detected the expression level of COX10-AS1 in clinical specimens collected during surgery and obtained a similar result. By analysis of the clinical data, we found that the expression level of COX10-AS1 was related to the prognosis and recurrence of patients with glioma. In addition, qRT-PCR demonstrated that the expression of COX10-AS1 in glioma cell lines was higher than that in NHAs, especially in U87 and LN229. To explore the function of COX10-AS1 in glioma, a series of gain- and loss-of-function assays were conducted. The results showed that downregulation of COX10-AS1 inhibited the proliferation, migration and invasion of glioma, whereas upregulation of COX10-AS1 caused the opposite effect. These findings indicated that COX10-AS1 plays a key role in glioma progression, which prompted us to investigate the potential mechanism.

By the ceRNA mechanism, a lncRNA can act as a sponge for miRNAs, leading to the latter being inactivated, thereby losing its regulatory effect on the targeted mRNAs (27). Recently, the ceRNA mechanism has been shown to be widely involved in human cancers. For example, Wang et al. indicated that NEAT1 aggravates endometrial cancer progression by sponging miR-144 (28); Yuan et al. reported that linc00994 is involved in the proliferation and invasion of gastric cancer by sponging miR-765-3p (29); and Liu et al. showed that HOTAIR acts as a sponge for miR-126 to regulate glutaminase in glioma (18). In the current study, the information from StarBase and the experiments we performed indicated that COX10-AS1 could sponge miR-641. qRT-PCR showed that miR-641 was downregulated in glioma and was correlated with glioma recurrence, which indicated that miR-641 may be involved in the regulation of COX10-AS1 in glioma. Previous studies have demonstrated that miR-641 participates in the development of some tumours. For instance, Kong et al. reported that miR-641 inhibits the progression of lung cancer (30); Chen et al. showed that miR-641 is involved in the erlotinib resistance of non-small-cell lung cancer (31); and Yao et al. suggested that miR-641 acts as a tumour suppressor in cervical cancer (32). There is also a study that reported that miR-641 could target ATK2 to regulate glioma progression (33); however, the role and mechanism of miR-641 in glioma still need further study. In this research, we found that, consistent with a previous study, miR-641 was downregulated in glioma. Moreover, miR-641 could partly inhibit the promoting effect of COX10-AS1 on glioma progression.

To test our scientific hypothesis, we selected the target mRNAs of miR-641 from an online database. From these candidate genes, we found that E2F6 was upregulated in glioma tissues and cell lines, which was closely related to COX10-AS1 and miR-641. As a well-known member of the E2F family, E2F6 plays a critical role in human disease. Li et al. reported that E2F6 regulates gastric carcinoma development by targeting CASC2 (34); Shi et al. suggested that E2F6 acts as a target of TLX to regulate islet beta cell proliferation (35); and Cai et al. documented that E2F6 is involved in miR-425-mediated growth renal cell carcinoma (36). However, the role of E2F6 in glioma has never been reported. It is imperative to investigate the role and underlying mechanism of E2F6 in glioma. Through a series of assays, we confirmed that E2F6 is a functional target of the COX10-AS1/miR-641 axis to regulate proliferation, migration and invasion.

**Figure 10:**
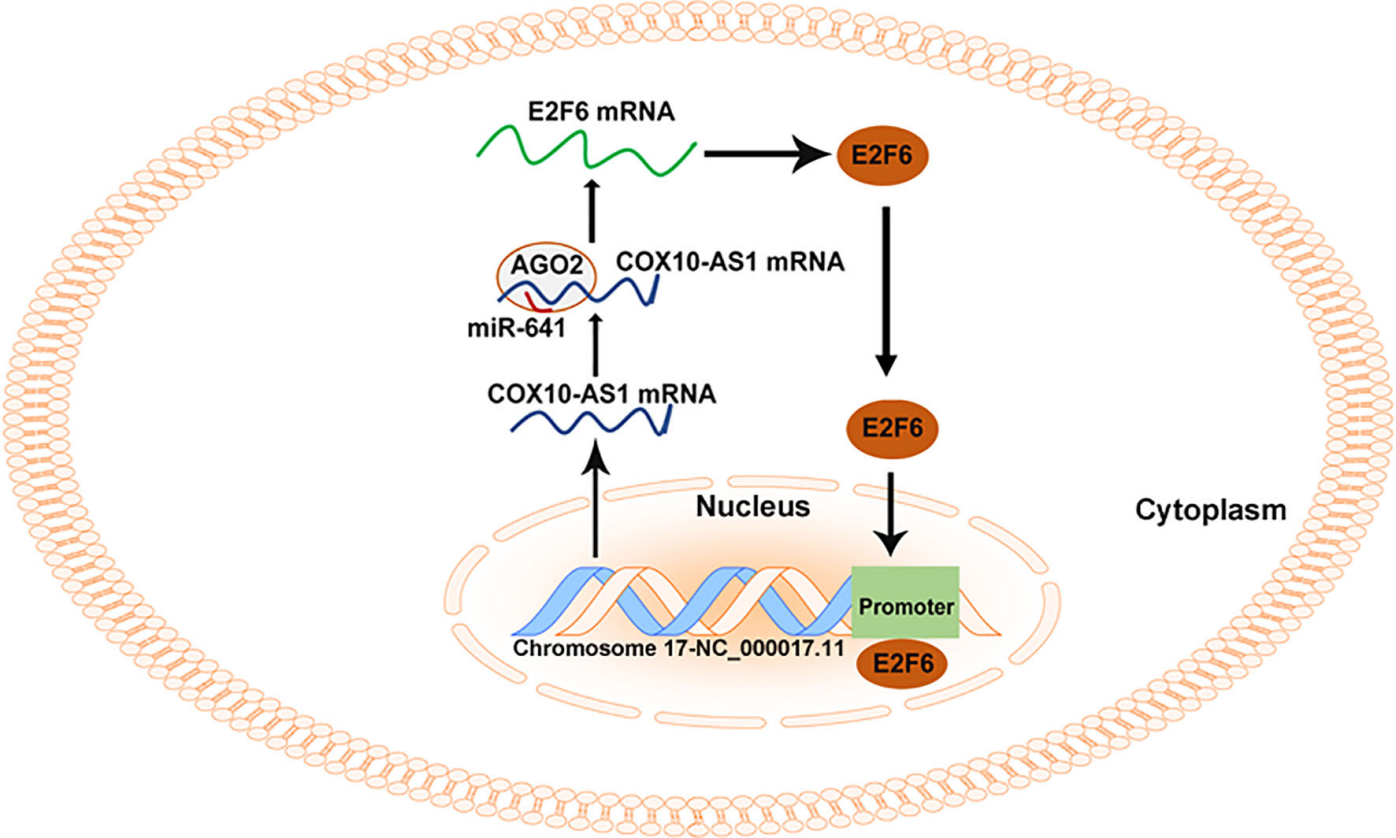
Summary of the COX10-AS1/miR-641/E2F6 feedback loop in glioma progression.

As COX10-AS1 is a ncRNA, there is no doubt that the transcription process of COX10-AS1 is regulated by transcription factors. E2F6 is a well-known transcription factor, and we were interested in whether E2F6 could regulate the transcription process of COX10-AS1, which is of great significance to further study the mechanism of COX10-AS1/miR-641/E2F6 in glioma. Through a literature search, we found that there is no research on the regulatory relationship between E2F6 and COX10-AS1. To address this issue, we used the JASPAR database. According to the prediction from JASPAR, there were existing sites in the promoter of COX10-AS1 to which E2F6 could bind. The effective binding sites of E2F6 on COX10-AS1 were verified by luciferase reporter assay and ChIP. In addition, the results of qRT-PCR and western blotting showed that downregulation of COX10-AS1 could repress E2F6 expression and that E2F6 could promote the transcription of COX10-AS1. These results suggested that COX10-AS1/miR-641/E2F6 formed a positive feedback loop to regulate glioma progression (**Figure 10**).

Taken together, in present study, we showed that E2F6-induced COX10-AS1 promotes glioma progression by acting as a sponge for miR-641 to regulate E2F6. These findings indicated that the E2F6/COX10-AS1/miR-641 feedback loop plays an important role in glioma and may be considered a potential therapeutic target for glioma patients.

## Data Availability Statement

The raw data supporting the conclusions of this article will be made available by the authors, without undue reservation.

## Ethics Statement

The studies involving human participants were reviewed and approved by Ethics Committee of the Nanjing First Hospital. The patients/participants provided their written informed consent to participate in this study. The animal study was reviewed and approved by Ethics Committee of the Nanjing First Hospital.

## Author Contributions

LL performed the cell functional experiments, analyzed the data, and prepared the figures. XiaoL performed the experiments and wrote the manuscript. HW designed the experiments and collected clinical specimen. YT performed the animal experiments. XianL discussed the manuscript. YS designed the experiments, supervised the research and revised the manuscript. All authors contributed to the article and approved the submitted version.

## Funding

This study was supported by grants from National Natural Science Foundation of China (No. 81502168) and Nanjing Medical Science and technique Development Foundation.

## Conflict of Interest

The authors declare that the research was conducted in the absence of any commercial or financial relationships that could be construed as a potential conflict of interest.

## Publisher’s Note

All claims expressed in this article are solely those of the authors and do not necessarily represent those of their affiliated organizations, or those of the publisher, the editors and the reviewers. Any product that may be evaluated in this article, or claim that may be made by its manufacturer, is not guaranteed or endorsed by the publisher.
