# Supplementary material for: The COX10-AS1/miR-641/E2F6 Feedback Loop Is Involved in the Progression of Glioma
Source: Front Oncol. 2021 Jul 26;11:648152. doi: 10.3389/fonc.2021.648152 (PMC8350443; doi:10.3389/fonc.2021.648152)
Supplement: Supplementary file 1 [file Table_1.docx]

**Table S1:** **The interfering nucleotide used in this study**

| **Name** | **Sequences** |
| --- | --- |
| sh-COX10-AS1 NC | 5′-GGCTTTGCAAATGCGATAGCCGT-3′ |
| sh-COX10-AS1-1 | 5′-TTGGCAGCAACGTAGTGCGATGA-3′ |
| sh-COX10-AS1-2 | 5′-GGCGTAGTGAAATGAAACGTAAA-3′ |
| miR-641 mimic | 5′-GACGAUCCUCUGCAUCGAGUA-3′ |
| miR-641 mimic NC | 5′-UAGUUGACGCUUGACGCUAGU-3′ |
| miR-641 inhibitor | 5′-GAUCGGACAUGGUGCUAGCUU-3′ |
| miR-641 inhibitor NC | 5′-AGUCGACGAUAUGCCAGGUCG-3′ |
| sh-E2F6 NC | 5′-AGCGCGAGTATACGTGCCTA-3′ |
| sh-E2F6 | 5′-GCGCTTGGGATCTAGGTTCA-3′ |
