# Supplementary material for: The COX10-AS1/miR-641/E2F6 Feedback Loop Is Involved in the Progression of Glioma
Source: Front Oncol. 2021 Jul 26;11:648152. doi: 10.3389/fonc.2021.648152 (PMC8350443; doi:10.3389/fonc.2021.648152)
Supplement: Supplementary file 2 [file Table_2.docx]

**Table S2: The primers used in this study**

| **Gene name** | **Sequences** |
| --- | --- |
| COX10-AS1 | F: 5′-TATCGAACGGTACTTGCTTACG-3′ |
|  | R: 5′-TGGCTAGTGACCCGGTAGTCA-3′ |
| miR-641 | F: 5′-TTATACTCTCACCATTTGGATC-3′ |
|  | R: 5′-TTATACTCTCACCATTTGGATC-3′ |
| E2F6 | F: 5′-GACCTCGTTTTGATGTATCGCTG-3′ |
|  | R: 5′-ATACACTCTCCGCTTTCGGAC-3′ |
| β-actin | F: 5′-CCACGAAACTACCTTCAACTCC-3′ |
|  | R: 5′- GTGATCTCCTTCTGCATCCTGT -3′ |
